# Introducing *Communications Biology*

**DOI:** 10.1038/s42003-017-0012-4

**Published:** 2018-01-22

## Abstract

We are pleased to introduce *Communications Biology*. Our aim is simple: to provide a place for all biologists, regardless of research topic, to publish high-quality work that advances their field of research.

Today marks the official launch of *Communications Biology*. We are thrilled to publish the first of what will be many articles reporting exciting advances across the diverse spectrum of disciplines within the life sciences.

Why are we launching this journal, at this time? Biologists already have a dizzying number of journals to select from when choosing where to submit their latest research. At the same time, the number of research articles published each year continues to grow and staying abreast of the literature has become a daunting, if not impossible, task for any researcher. We believe that journals have an important role to play in filtering the research to provide at least a rough guide to scientists for where they can find the latest research of most importance to their work. *Communications Biology* fills a specific need within the Nature Research portfolio for a high-quality, broad-scope biology journal publishing work of importance to fellow researchers without the need for articles to be of broad, general interest outside a specialist community—a theme that extends to our sister journals, *Communications Chemistry* and *Communications Physics*. It also fills a more general need as one of still only a small number of life sciences journals providing a broad platform for specialist advances.

Our title, *Communications Biology*, says a lot about what we hope to achieve. Our editors select research for peer review on the basis of whether it advances an area of inquiry within the biological sciences. However, the journal communicates that research not only to other specialists, but to all biologists. Through the publication of Reviews and Comments, we provide a place for biologists to communicate new ideas or announce new developments of importance to the community. *Communications Biology* also describes our publishing model. We are a fully open access, online only journal, allowing us to make our authors’ work immediately available so it can be communicated rapidly to all who wish to read it. And, finally, our editorial model is also set up to foster communication between our in-house editors and editorial board members, the latter of whom are active researchers in the community we aim to serve.

Our in-house editors and academic editorial board members all share the same passion for service to the community through editorial excellence.

As part of our goal to help biologists communicate their research, we strive to provide the highest possible level of editorial service, just as scientists have come to expect from the Nature Research journals. Our in-house editors and academic editorial board members all share the same passion for service to the community through editorial excellence. The editorial and review process with *Communications Biology* will be honest, transparent, and constructive, regardless of the final outcome. We sincerely hope that if we ever fall short of our goals, you—our readers, authors, and reviewers—will remind us of our mission. Communication cannot be a one-way street.

Our editorial focus is on helping biologists communicate their work to the world, whether it is translational research with obvious potential for impacting human health, or basic research that provides the raw material for future technological and medical breakthroughs. We believe this is especially important now, in these tumultuous times when the value of scientific inquiry to humanity is being questioned more than ever. We will not reject a paper for lack of applied utility as long as the research provides new biological insight. At the same time, we will consider innovative and useful tools and methods that will further advance biological or medical research in the future, without the need to demonstrate a new biological discovery.

Our first few papers offer a glimpse of the diversity of biology that we will publish, from a study of environmental DNA degradation in river systems (Seymour et al.), to a molecular mechanism of chromatin remodeler regulation (Turegun et al.) and an ultra-high-throughput method for antibody selection (Rajan et al.). The Review by Ryszard Maleszka invites us to consider the complexity of developmental and epigenetic programs in the determination of honeybee castes. And we will be publishing across a wider range of subject areas as we grow. Our editors are committed to serving the needs of each unique community of biologists—ecologists, evolutionary biologists, molecular biologists, geneticists, neuroscientists, plant scientists, microbiologists, biophysicists, biochemists, computational biologists—and those biologists who defy labels.

Today is only the beginning of a long journey. We hope you will join us on this journey and help contribute to the future success of *Communications Biology*.

